# COVID-19 in Pediatric Patients: A Study Based on Biomarker Levels

**DOI:** 10.7759/cureus.39408

**Published:** 2023-05-23

**Authors:** Walaa Mohammedsaeed, Fahad Alsehli, Lutfi Alfarsi, Ameen Bakhsh, Mansour Alzahrani, Maram Almarwani, Yousef Alharbi

**Affiliations:** 1 Diabetes and Endocrinology, Clinical Biochemistry, Taibah University, Madinah, SAU; 2 Pathology, King Salman Medical City, Madinah, SAU; 3 Pathology, Medina Regional Laboratory, Madinah, SAU; 4 Laboratory, Maternity and Children Hospital, Madinah, SAU; 5 Laboratory, Poison control and Forensic Chemistry Center, Madinah, SAU

**Keywords:** infection, sars-cov-2, biochemical markers, pediatric patients, severity of covid-19

## Abstract

Background: Severe acute respiratory syndrome coronavirus 2 (SARS-CoV-2) has infected people of all ages, but limited data are available on children with mild and severe coronavirus disease 2019 (COVID-19).

Methods: Clinical characteristics, inflammation, and other biochemical biomarkers have been described, but information is scarce in asymptomatic and mild cases. Laboratory investigations were performed with pediatric patients (n=70) for liver function and kidney function, along with C-reactive protein (CRP).

Results: Mild clinical characteristics and symptoms were observed in pediatric patients. Even in moderate cases of COVID-19, elevated levels of biomarkers indicate altered liver and kidney function in children. The levels of liver enzymes, bilirubin, creatinine, and CRP varied significantly between the three classes, particularly between asymptomatic and moderate cases. Liver enzymes, bilirubin, and creatinine levels in moderate COVID-19 pediatric cases were twice as elevated as in asymptomatic cases. Liver enzymes and CRP levels were moderately elevated.

Conclusion: Monitoring blood biomarkers consistently can assist in the accurate identification of infection in young patients as well as in the prevention of its spread and the administration of appropriate treatment.

## Introduction

Coronavirus disease 2019 (COVID-19 is an ongoing pandemic since March 11, 2020, and until the time of writing this article (August 2022), there have been about 660 million people affected and 6.7 million killed globally [1]. People of all ages are vulnerable to this infection, but children seem to be specifically less susceptible [2,3]. Besides, in comparison to adult patients, children presented milder symptoms [4]. For reasons not clearly understood, many children remain asymptomatic or mildly symptomatic and hence remain undiagnosed. They prove to be major carriers of viruses, unknowingly transmitting them to other vulnerable groups of people. Understanding the role of children in viral transmission and their response to the infection is of utmost concern now that schools have reopened all over the world, although COVID-19 cases are still emerging. Reports also suggest that children are mainly infected after coming into contact with other family members at home [5]. Recent reports indicate that there were 518,057 pediatric cases (between 0 and 19 years of age) in Italy, representing 14.1% of the affected population, with 22 pediatric fatalities. Infection with SARS-CoV-2 in children is uncommon, typically spreads within family clusters, and manifests with mild and diverse symptoms [6-8], including fever, nasal congestion, cough, dyspnea, myalgia, arthralgia, headache, gastrointestinal, and skin manifestations with the characteristic anosmia and ageusia [8,9]. Rarely, severe respiratory symptoms necessitating intensive care, or multisystem inflammatory syndrome (MIS-C) have been reported [10,11].

Furthermore, the disease has spread to numerous regions in Saudi Arabia. There have been 377,383 confirmed cases, of which 2.22% have been fatal [12,13]. Children account for 1% to 5% of all cases of COVID-19 and are less likely to report symptoms [13,14]. Based on Saudi Arabian data indicating an incidence of approximately 8%, the preponderance of pediatric cases is identified through contact tracing [15]. Infants younger than one year are the most susceptible to COVID-19 infection among all pediatric age categories [16,17]. Currently, epidemiological data regarding pediatric COVID-19 disease is available, and several investigations have been conducted in Saudi Arabia [18-20]. To acquire a better understanding of the disease's nature and effects, however, additional research is required.

Since COVID-19 is a disease that affects multiple organs, several biochemical and other biomarkers can be used to understand the pathogenesis. Information regarding serum biomarker levels can help in timely diagnosis and appropriate treatment, and save the lives of critical patients. Biomarkers of COVID-19 can assist in the confirmation of disease severity as a deciding factor for hospital admission and treatment strategies [20- 21]. Studies have to be conducted to comprehend the differential character of biomarkers in the advancement of disease in both adult and pediatric patients. In general, children with abnormalities show severe illness; otherwise, only mild symptoms are displayed. Laboratory biochemical biomarkers have been described in COVID-19 for liver function (alkaline phosphatase (ALP), alanine transferase (ALT), aspartate transferase (AST), gamma-glutamyl transferase (GGT), bilirubin) and kidney function (blood urea nitrogen (BUN), chloride (Cl), creatinine, sodium (Na), glucose, potassium (K)). Other markers like levels of calcium (Ca), magnesium (Mg), albumin, total proteins, and C-reactive protein (CRP) are also good indicators [22,23]. The purpose of the present study was to evaluate the biomarker levels in children with COVID-19 while comparing the levels in adults during the infection period. The association between the biomarker alteration and the severity of the disease was also analyzed. Identification of biomarkers in infected children can help physicians provide suitable treatment for children at the earliest and also provide data for the development of COVID-19-appropriate guidelines specific to children.

## Materials and methods

This retrospective research was carried out at the Maternity and Children's Hospital, Madinah, Saudi Arabia. In June 2021, 70 cases of COVID-19 infection in children aged three months to 13 years were selected at random. In this context, the word "sampling random" refers to the population from which samples are collected; in this situation, it would be a list of all pediatric patients who are relevant to the research. We focused on children who were diagnosed with COVID-19 infection throughout the research period (2021). Following their entry into a database, the clinical and laboratory data of the patients were analyzed before being interpreted. The inclusion and exclusion criteria were applied to each and every one of the instances that were examined. Patients who met the inclusion criteria of having confirmed COVID-19 were further divided into asymptomatic, mild, moderate, and severe categories based on their clinical characteristics, symptoms, and chest radiography results [20]. As patient information had been carefully recorded in the respective patient files, a database capable of being examined was developed.

Alterations in biomarker levels were assessed, and the clinical relevance of organ function was investigated in the laboratories of the hospital. By assessing biomarkers for liver function (including ALP, ALT, AST, GGT, and bilirubin) and kidney function (including BUN, Cl, creatinine, Na, glucose, and K), the researchers investigated their association with the progression of the illness.

For the analysis of the data, the Statistical Package for the Social Sciences (SPSS) version 20 (IBM Corp., Armonk, NY USA) or GraphPad Prism 7 (GraphPad Software, San Diego, CA, USA) was used. Quantitative data is stated in a variety of ways, including percentages, the mean, the standard deviation (SD), and the range. In order to evaluate the degree of similarity or difference between the results obtained from the various study groups, a two-way analysis of variance (ANOVA) was carried out. All of the differences were determined to be statistically significant when compared to a threshold of p≤0.05 or 0.001, respectively. This study was approved by the Institutional Review Board of Maternity and Children Hospital-Madinah (approval no. 100-2021).

## Results

General and clinical characteristics of children with SARS-CoV-2 infection

For the present study, COVID-19-positive children (n=70; 28 boys and 42 girls) were selected on the basis of RT-PCR analysis from Maternity and Children Hospital in Madinah during the period of study (2021) The youngest child was a three-month-old male and the oldest was a 13-year-old female (mean age was 10±4.8 years). All the children had a mild fever (37.4^°C^ to 38.5^°C^) with a mild cough and a sore throat. None of the children needed intensive care, mechanical ventilation and did not have any severe complications. The biochemical estimations revealed that children had increased levels of AST (25± 10 U/L), GGT (32± 11.4 U/L), BUN (20± 1.8 mmol/L), bilirubin (41± 27 µmol/L), Cl (136± 26 mmol/L), and CRP (25± 8.5 mg/L) but lower levels of Na (52± 38 mmol/L) in comparison to the normal range. The total protein content at 25± 3.4 g/L was also lower than the normal range (Table 1).

**Table 1:** General and clinical Characteristics and laboratory blood test results for children with SARS-CoV-2 infection Data presented as mean ± SD for age and biomarkers levels in children with SARS-CoV-2 infection AST: Aspartate aminotransferase, ALT: Alanine aminotransferase, ALP: Alkaline phosphatase, GGT: Gamma glutamyl-transferase, BUN: Blood urea nitrogen, Na: Sodium, K: Potassium, P: Phosphorus, C: Calcium, Cl: Chloride, CRP: C-reactive protein

Parameters	Children with COVID-19 (n=70)	Normal range
Age (mean ± SD)	10 ± 4.8	-
Gender (Number)	Male=28, Female=42	-
History of diseases	None	-
Symptoms	Mild fever (37.4-38.5°C), Mild cough, Sore throat	
Glucose (mmol/L)	6± 1.5	3.9-7.1 mmol/L
ALT (U/L)	21± 18	24-49 U/L
AST (U/L)	25± 10	1-3.6 U/L
ALP (U/L)	97± 37	44- 147 U/L
GGT (U/L)	32± 11.4	9-20 U/L
Total protein (g/L)	25± 3.4	63-81 g/L
Bilirubin (µmol/L)	41± 27	0-14 µmol/L
Albumin (g/L)	31± 18.3	38-56 g/L
Creatinine (µmol/L)	26± 17	20-70 µmol/L
Urea (mmol/L)	16± 7.5	5-18 mg/dL
BUN (mmol/L)	20± 1.8	1.6-4.6 mmol/L
Na (mmol/L)	52± 38	132-141 mmol/L
K (mmol/L)	5± 4.2	3.3-4.7 mmol/L
Ca (mmol/L)	3± 1.2	2.22-2.52 mmol/l
Cl (mmol/L)	136± 26	97-107 mmol/L
CRP (mg/L)	25± 8.5	0.02 to 14.4 mg/l

Alterations in biomarker levels in different illness classes in children

The patients were classified according to disease severity and symptoms. It was observed that 24.28% were asymptomatic, 34.28% were mild, and 41.4% were moderate. No severe cases were observed in the present study. The typical clinical manifestations were fever (55%) and cough (45%). As shown in Table 2, there were significant differences in the levels of ALT, AST, GGT, bilirubin, creatinine, and CRP between the three classes, especially between asymptomatic and moderate cases (p<0.05*, <0.001**). The ALT, bilirubin, and creatinine levels were two-fold higher in moderate COVID-19 pediatric cases than in asymptomatic cases. The AST, GGT, and CRP levels were only slightly higher (~1.5 fold) (Table 2).

**Table 2:** Biomarker levels in asymptomatic, mild, and moderate cases of COVID-19 in children Data were evaluated by a two-way ANOVA test to compare the three categories of  COVID-19 in pediatric patients. A statistically significant difference was considered as p<0.05* or <0.001** AST: Aspartate aminotransferase, ALT: Alanine aminotransferase, ALP: Alkaline phosphatase, GGT: Gamma glutamyl-transferase, BUN: Blood urea nitrogen, Na: Sodium, K: Potassium, P: Phosphorus, C: Calcium, Cl: Chloride, CRP: C-reactive protein

Biomarkers	Asymptomatic cases, n=17	Mild cases, n=24	Moderate cases, n=29	p-value
	Mean± SD	Mean± SD	Mean± SD	
ALT (U/L)	10± 5.8*	19± 9.7	20± 11	0.03*
AST (U/L)	15± 7.9*	20± 9.6	24± 13	0.02*
GGT (U/L)	22± 10.4*	30± 11.7	35± 15.4	0.03*
Total Protein (g/L)	22± 7.5	24± 8.9	26± 8.4	>0.05
Bilirubin (µmol/L)	20± 12.7*	43± 22	45± 25	0.04*
Creatinine (µmol/L)	16± 6.3*	24± 11	32± 14.3	0.01*
Urea (mmol/L)	11± 6.7	12± 6.5	14± 7.5	>0.05
BUN (mmol/L)	18.5± 1.8	19.3± 10.8	21± 11.8	>0.05
Na (mmol/L)	56± 33	52.7± 38	52.9± 39	>0.05
Cl (mmol/L)	130± 16	135± 20	137± 21	>0.05
CRP (mg/L)	18± 6.9*	28± 9.5	30± 10.5	0.004**

## Discussion

Pediatric COVID-19 patients display less severe symptoms, the reason for which needs to be investigated. A similar pattern has been observed earlier with other coronaviruses (SARS and Middle East respiratory syndrome (MERS)), where both the number and severity of the disease were low in children [20,24]. Of equal significance is understanding how the level of biomarkers is altered during the infection. The present study shows that the COVID-19-infected children had mild symptoms with no serious complications. The liver enzymes AST and GGT were only slightly elevated, while ALT levels were lower than the normal range (Table 1). Our results corroborated well with previous studies by Bourkhissi et al., Henry et al., and Hon et al. [22-24]. The BUN, bilirubin, and CRP levels were also higher, but levels of Na were lower than the normal range, indicating some decline in both liver and kidney function. The deviation from the normal range was not very drastic. Moreover, the higher levels may also be due to other reasons; for instance, elevated BUN may also be due to dehydration, an injury, or a high protein diet [24,25]. The creatinine level was within the normal range. The BUN-to-creatinine ratio is more specific than the BUN test alone. Severe acute respiratory syndrome coronavirus 2 can directly affect the kidneys and lungs by binding to angiotensin-converting enzyme (ACE2) receptors present in the cells of these vital organs [26]. 

Since children are mostly asymptomatic and free of underlying co-morbidities, diagnosis and infection source control are more problematic [27]. Around 30% of adult patients reach the ICU, while pediatric patients are occasionally admitted to the hospital [28]. Also, the duration of hospital stay is shorter [29]. Alterations in serum biomarker levels were more common in these children, maybe due to organ tissue damage as a result of the disease [30]. Systemic inflammation due to higher levels of IL-6 and IL-10 was weaker in pediatric patients (mild to moderate cases). The CRP, a serum inflammatory marker protein, showed abnormal levels in children with COVID-19 and has been identified as a probable biomarker for disease severity [30]. The reason for lower infection rates in children can be manifold. During the pandemic, children were confined to their homes with no school and outdoor activities. Whatever minimal exposure they had was from adults at home.

The SARS-CoV-2, an RNA virus, may be susceptible to mutations, incorrect replication, and immune evasion. As a result, it can be suggested that children have been exposed to later-generation viruses with reduced virulence. Hence, milder symptoms may be observed due to significant differences in the immune response triggered in children in comparison to adults. The innate immune response (first line of defense) is more robust in children and is capable of taking care of pathogens more swiftly. On the contrary, the adaptive immune response (antibody generation by B lymphocytes) is activated when innate immunity is insufficient. It is more specific but takes longer to recognize the pathogen [29,30]. It is well known that the virus enters the host with the help of ACE2 receptors and transmembrane serine protease 1 (TMPRSS2) [30]. The number, distribution, and function of these receptors are different in children and adults, hence the difference in severity of the disease and symptoms.

Another important factor is that the general health status of children and their habits are much better than those of adults. For example, there are more smokers, alcohol, and tobacco consumers in older people [30]. A comparison of biomarkers estimated in COVID-19 patients showed that ALT, AST, bilirubin, urea, BUN, and Cl levels were significantly higher in children than in adults, but the levels of total protein, creatinine, Na, and CRP were lower in children in comparison to adult patients. Of all the biochemical parameters, bilirubin levels were significantly higher (29-fold) in children. Bilirubin accumulation is the result of the liver losing its ability to excrete it from the blood, and such high levels indicate that the liver has been affected significantly. Studies suggest that COVID-19-associated liver injury is accompanied by an insignificant increase in transaminases, which is also corroborated by the present work [28-30].

The limitations of the present study are that it only included patients from a single hospital and that the sample size was small. Nevertheless, the study is significant as it can help in the identification and diagnosis of disease patterns in children with mild to moderate infection. A comparative analysis of samples from other hospitals will give a clearer representation.

## Conclusions

Children in particular need to be screened based on organ damage indicators that may be quickly found before the patient develops a major disease if they are just exhibiting moderate symptoms of COVID-19. In addition to the evaluation of other clinical indicators, testing for liver and kidney function may help in the process of identifying asymptomatic individuals, which is important in order to quickly contain the virus and halt its spread. It is necessary to perform more studies with more samples and a wider range of biomarkers.

## References

[1] (2021). WHO Coronavirus (COVID-19) Dashboard | WHO Coronavirus (COVID-19) Dashboard With Vaccination Data. https://covid19.who.int/.

[2] Ding Y, Yan H, Guo W (2020). Clinical characteristics of children with COVID-19: a meta-analysis. Front Pediatr.

[3] Cui X, Zhao Z, Zhang T (2021). A systematic review and meta-analysis of children with coronavirus disease 2019 (COVID-19). J Med Virol.

[4] Galindo R, Chow H, Rongkavilit C (2021). COVID-19 in children: clinical manifestations and pharmacologic interventions including vaccine trials. Pediatr Clin North Am.

[5] Su L, Ma X, Yu H (2020). The different clinical characteristics of coronavirus disease cases between children and their families in China—the character of children with COVID-19. Emerg Microbes Infect.

[6] Brindisi G, De Vittori V, De Nola R (2021). Updates on children with allergic rhinitis and asthma during the COVID-19 outbreak. J Clin Med.

[7] Chan JF, Yuan S, Kok KH (2020). A familial cluster of pneumonia associated with the 2019 novel coronavirus indicating person-to-person transmission: a study of a family cluster. Lancet.

[8] Eastin C, Eastin T (2020). Epidemiological characteristics of 2143 pediatric patients with 2019 coronavirus disease in China. J Emerg Med.

[9] Parisi GF, Brindisi G, Indolfi C (2020). Upper airway involvement in pediatric COVID-19. Pediatr Allergy Immunol.

[10] Gori A, Leone F, Loffredo L (2020). COVID-19-related anosmia: the olfactory pathway hypothesis and early intervention. Front Neurol.

[11] Kabeerdoss J, Pilania RK, Karkhele R, Kumar TS, Danda D, Singh S (2021). Severe COVID-19, multisystem inflammatory syndrome in children, and Kawasaki disease: immunological mechanisms, clinical manifestations and management. Rheumatol Int.

[12] AlGhamdi A, Al Talhi Y, Al Najjar A (2022). Epidemiology, clinical characteristics and risk factors of COVID-19 among children in Saudi Arabia: a multicenter chart review study. BMC Pediatr.

[13] (2022). Daily Updates - Public Health Authority. https://covid19.cdc.gov.sa/daily-updates/.

[14] CDC COVID-19 Response Team (2020). Coronavirus Disease 2019 in children - United States, February 12-April 2, 2020. MMWR Morb Mortal Wkly Rep.

[15] Zimmermann P, Curtis N (2020). Coronavirus infections in children including COVID-19: an overview of the epidemiology, clinical features, diagnosis, treatment and prevention options in children. Pediatr Infect Dis J.

[16] Alyami MH, Naser AY, Orabi MA, Alwafi H, Alyami HS (2020). Epidemiology of COVID-19 in the Kingdom of Saudi Arabia: an ecological study. Front Public Health.

[17] Dong Y, Mo X, Hu Y, Qi X, Jiang F, Jiang Z, Tong S (2020). Epidemiology of COVID-19 among children in China. Pediatrics.

[18] Alharbi M, Kazzaz YM, Hameed T (2021). SARS-CoV-2 infection in children, clinical characteristics, diagnostic findings and therapeutic interventions at a tertiary care center in Riyadh, Saudi Arabia. J Infect Public Health.

[19] Kari JA, Shalaby MA, Albanna AS (2021). Coronavirus disease in children: a multicentre study from the Kingdom of Saudi Arabia. J Infect Public Health.

[20] (2021). MOH-approved scientific instruction manuals and guidelines for healthcare providers on how to deal with COVID-19 patients. https://www.moh.gov.sa/Ministry/MediaCenter/Publications/Documents/MOH-therapeutic-protocol-for-COVID-19.pdf.

[21] Samprathi M, Jayashree M (2020). Biomarkers in COVID-19: an up-to-date review. Front Pediatr.

[22] Bourkhissi L, Fakiri KE, Nassih H (2020). Laboratory abnormalities in children with novel coronavirus disease 2019. Clin Med Insights Pediatr.

[23] Henry BM, Benoit SW, de Oliveira MH (2020). Laboratory abnormalities in children with mild and severe coronavirus disease 2019 (COVID-19): a pooled analysis and review. Clin Biochem.

[24] Hon KL, Leung CW, Cheng WT (2003). Clinical presentations and outcome of severe acute respiratory syndrome in children. Lancet.

[25] Delorey TM, Ziegler CG, Heimberg G (2021). COVID-19 tissue atlases reveal SARS-CoV-2 pathology and cellular targets. Nature.

[26] Dhochak N, Singhal T, Kabra SK, Lodha R (2020). Pathophysiology of COVID-19: why children fare better than adults?. Indian J Pediatr.

[27] Sinaei R, Pezeshki S, Parvaresh S, Sinaei R (2021). Why COVID-19 is less frequent and severe in children: a narrative review. World J Pediatr.

[28] Qian JY, Wang B, Liu BC (2020). Acute Kidney Injury in the 2019 novel coronavirus disease. Kidney Dis (Basel).

[29] Li B, Zhang S, Zhang R, Chen X, Wang Y, Zhu C (2020). Epidemiological and clinical characteristics of COVID-19 in children: a systematic review and meta-analysis. Front Pediatr.

[30] Tang C, Zhang K, Wang W (2020). Clinical characteristics of 20,662 patients with COVID-19 in mainland China: a systemic review and meta-analysis. medRxiv.

